# Investigation of accumulation of element contents in some wild and cultivated dried fruits

**DOI:** 10.1007/s12011-024-04165-w

**Published:** 2024-04-03

**Authors:** Fahad AlJuhaimi, Duygu Akçay Kulluk, Isam Ali Mohamed Ahmed, Mehmet Musa Özcan, Emad Karrar

**Affiliations:** 1 Department of Food Science & Nutrition, College of Food and Agricultural Sciences, King Saud University, Riyadh, Saudi Arabia; 2 Faculty of Agriculture, Department of Soil Science and Plant Nutrition, Selcuk University, 42031 Konya, Turkey; 3 Faculty of Agriculture, Department of Food Engineering, Selcuk University, 42031 Konya, Turkey; 4 College of Ocean Food and Biological Engineering, Jimei University, Xiamen, 361021 China

**Keywords:** Wild and cultivated fruits, Element accumulation, ICP-OES

## Abstract

In this study, the moisture and biogenic element quantities of most wild and cultivated edible dried fruits were investigated. Macroelements found in highest amounts in fruits were K, P, Ca, and Mg. While K quantities of the fruits are recorded between 5212.77 (“white myrtle”) and 25550.60 mg/kg (“*black nightshade*”), P amounts of the fruits were characterized to be between 949.08 (black myrtle) and 4420.75 mg/kg (“*black nightshade*”). Ca and Mg amounts of the fruits were assessed to be between 359.83 (plum) and 4330.89 mg/kg (“yellow hawthorn”) to 214.98 (plum) and 1852.04 mg/kg (“*black nightshade”*), respectively. Fe and B quantities of the fruitss were established to be between 2.69 (“black myrtle”) and 60.13 mg/kg (cherry) to 3.76 (“black myrtle”) and 76.25 mg/kg (sour cherry), respectively. In general, except for white and black myrtle fruits, it is thought that other fruits can be good sources of P, K, and Fe. P contents of “laurel cherry,” “cherry,” “black nightshade,” and “Fragrant black grapes” were found partly high than those of other fruits. “Cherry laurel,” “sour cherry,” “cherry,” “pear,” “black nightshade,” “black fig,” and “hawthorn (yellow)” fruits contain more K than other fruits.

**Graphical Abstract:**

In this study, the degree of accumulation of macro-, micro element contents of some wild and culktivated fruits was investigated.

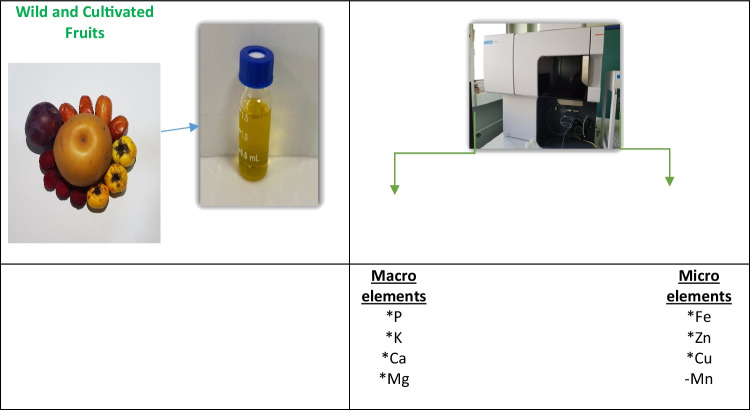

## Introduction

Fruits, which are important sources of nutrients, are often known as a good source of nutrients and food supplements in the world [13, 15]. Mineral ions are very important for human health, and potassium, calcium, and magnesium, found in the tissues of many fruits, have been reported to be an important factor determining the quality of fruit storage [3, 10, 16]. Fruits are rich in various fat-free components such as vegetable protein, fiber, nutrients, and bioactive compounds [26]. Dried fruits, which have high levels of antioxidants, vitamins, essential amino acids, nutrients, and phytochemicals, are very beneficial for human health [11, 12, 14, 18, 24]. Dried fruits have many medicinal properties because they contain plenty of nutrient compounds, and they should always be included in our diet for their health benefits [5]. The mineral content of a given food item has been reported to be an important factor characterizing the nutritional quality of the food product [21]. The main factors affecting the availability of mineral elements in the soil are soil pH, cation exchange capacity, activity of microbes, organic matter, and water content [7, 8]. The gradual increase in industrial and agricultural activities since the beginning of the twentieth century and the development of technologies accordingly have brought about some problems such as environmental pollution and deterioration of the world ecosystem balance, thus causing increasing contamination of foodstuffs [23, 9]. This situation has also threatened the health of all living creatures and endangered the sustainability of the ecological environment. In recent years, heavy metals have emerged as one of the most important food contaminants that threaten human health [25]. Since a large-scale study determining the biogenic element contents of dried fruits is rare, the necessary amounts of these elements must be in the human diet to maintain a healthy life [19]. It can be said that these fruits, which have high nutritional value and important usage areas, are not known and do not receive the necessary attention in many regions of our country. Products such as pulp, nectar, marmalade, tea, and fruit extract can be obtained from these wild fruit species. In addition to enriching other fruit and vegetable juices with vitamins, it can also be used as a filling material in the cake and confectionery industry [4]. The aim of this research was to determine the macro and micro element contents of wild and cultivated edible fruits obtained from 4 different locations after drying.

## Material and Methods

### Material

**Fig. 1:**
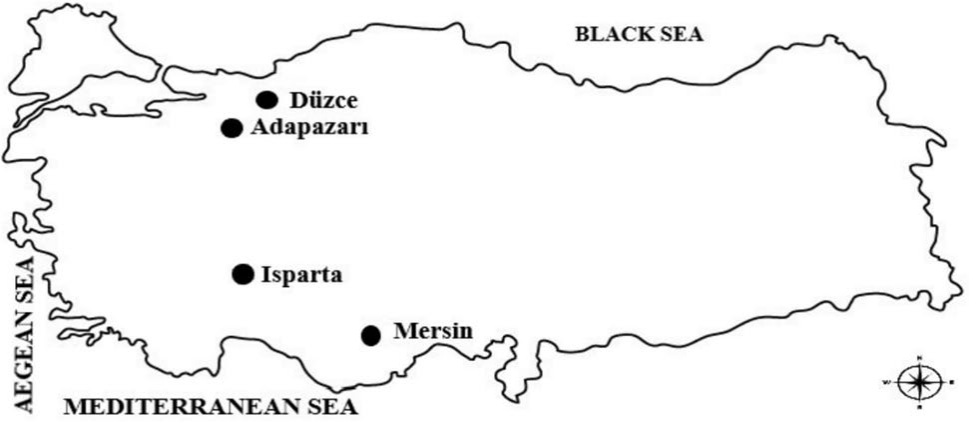
Locations where the fruits used in this study were collected

Fruit samples were provided from Düzce (“Cherry Laurel,” “Fragrant Black Grapes,” “Strawberry Tree,” “Hawthorn (Yellow),” “Japonica Pear”), Adapazarı (“Fragrant black Nightshade,” “Kaki Persimmon,” “Black Fig,” “Damask Plum”), Isparta (“Sour Cherry,” “Cherry,” “Elaeagnus”), and Mersin (“White and black myrtles”) provinces in 2023 (Table 1). These fruits were preferred because they were more abundant in these locations. After the fruits were collected, they were transported to the laboratory in cold bags. Before the analysis, the fruits were washed with distilled water, and then the dried in a shade place. The environmental conditions are 68%, 32 °C, and 1100 m for average humidity, temperature, and altitude, respectively. HNO_3_ and H_2_O_2_ are analytical grade and Merck company (Darmstadt, Germany). The places where the fruits used in this study were collected are shown on the map below (Fig. 1).

**Table 1:** Names of fruit varieties and their Latin names and the parts where they are used

English name	Latin name	Used part	Type
Cherry laurel	*Prunus laurocerasus*	Flesh	Wild
Sour cherry	*Prunus cerasus* L	Flesh	Wild
Cherry	*Prunus avium*	Flesh	Wild
Pear	*Pirus communis*	Flesh	Wild
Common nightshade	*Solanum nigrum*	Flesh	Wild
Fragrant black grapes	*Vitis labruska*	Flesh	Wild
Kaki persimmon	*Diospyros kaki*	Flesh	Wild
Black fig	*Ficus carica*	Flesh	Wild
Strawberry tree	*Arbutus unedo*	Flesh	Wild
Hawthorn (yellow)	*Crataegus tanacetifolia*	Flesh	Wild
Elaeagnus	*Elaeagnus* spp.	Flesh	Culture
Damask plum	*Prunus domestica* L	Flesh	Culture
Japonica pear	*Pyrus communis*	Flesh	Wild
Myrtle (white)	*Myrtus communis* L	Flesh	Cultured
Myrtle (black)	*Myrtus communis* L	Flesh	Wild

### Method

#### Determination of Moisture

The moisture amounts of the fruits were assigned at 70 °C/48 h using an oven till a constant weight [2].

####   Macro and Micro- Element Contents of Fruit Samples

After 0.2 g ground fruit samples (0.5 meh) were inginerated in a microwave device at 210 °C and 200 PSI pressure /45 min in 5 ml of concentrated HNO_3_ and 2 ml of H_2_O_2_ (30% w/v), the volumes of the dissolved samples were completed to 20 ml with deionized water. Then, heavy metal concentrations in the samples were analyzed with Inductively coupled plasma-optical emission spectrometry (ICP-OES) [24].

#### Working Conditions of ICP-OES

Its RF power of ICP-OES changes between 0.7 and 1.5 kw (1.2–1.3 kw for axial). In addition, plasma gas flow rate (Ar) ranged from 10.5 to 15 L/min (radial) 15″ (axial). Auxilary gas flow rate (Ar) is 1.5″. Viewing height is between 5 and 12 mm. Copy and reading time change between 1 and 5 s (max. 60 s).

### Statistical Analysis

The JMP statistical program was used for the statistical analysis of results obtained. Statistically differences were determined by the analysis of variance (ANOVA) procedure in all data (*p* < 0.01) [22]. In order to examine the correlation between nutrient element contents of fruit types, a multivariate cluster analysis was carried out using the PAST statistical program to perform principal component analysis (PCA) [17].

## Results and Discussion

Macro and micro element quantities of cultivated and wild fruits obtained from different locations are depicted in Table 2. The element quantities of fruits showed some changes based on the fruit types. The macroelements detected in the highest amounts in the fruits studied were K, P, Ca, and Mg. While K quantities of the fruits are recorded to be between 5212.77 (“white myrtle”) and 25,550.60 mg/kg (“*black nightshade*”), P quantities of the fruit samples were characterized to be between 949.08 (“black myrtle”) and 4420.75 mg/kg (“*black nightshade*”). Calcium and Mg quantities of the fruits were recorded to be between 359.83 (“plum”) and 4330.89 mg/kg (“yellow hawthorn”) to 214.98 (“plum”) and 1852.04 mg/kg (“*black nightshade*”), respectively. Phosphorus, K, and Mg contents of Black Nightshade wild fruits were found to be much higher than those of cultivated fruits. The most abundant microelement detected in cultivated and wild fruits was B, followed by Fe, Mn, Cu, and Zn (Table 2). Iron and B results of the fruits samples were established to be between 2.69 (“black myrtle”) and 60.13 mg/kg (cherry) to 3.76 (“black myrtle”) and 76.25 mg/kg (“sour cherry”), respectively. While Zn amounts of the fruits vary between 0.84 (“black grape”) and 8.97 mg/kg (“*black nightshade*”), Mn amounts of the fruits changed to be between 1.83 (“elaeagnus”) and 12.59 mg/kg (“*black nightshade*”). In addition, Cu values of the fruit samples were assigned to be between 0.94 (“plum”) and 9.40 mg/kg (“*black nightshade*”). In general, except for “white and black myrtle” fruits, it is thought that other fruits can be good sources of P, K, and Fe. Phosphorus contents of “chery laurel,” “cherry” (“*black nightshade*”), and “black grape” were partly higher than other fruits. The highest K was detected in *black nightshade*, “cherry,” “black grape,” “black fig,” and “sour cherry” fruits. The fruits with the highest Ca amount were “hawthorn,” “cherry laurel,” “cherry” and “black myrtle.” While the highest Mg was detected in “*black nightshade*” and “black fig” fruits, the Mg amounts of other fruits were determined at very low levels. There were significant changes in the Fe content of the fruits, and the lowest Fe was determined in black myrtle, white myrtle, and “elaeagnus” fruits. Zinc contents of “sour cherry,” “cherry,” “*black nightshade*,” “black fig,” “hawthorn,” “elaeagnus,” and “black myrtle” were higher than those of other fruits. The B absorption ability of cherry laurel, sour cherry, black grape, and cherry was found to be higher than that of other fruits. In general, among the fruits studied, the fruit containing the highest P, K, Mg, Zn, Cu, and Mn was the currant fruit. In previous study, Fe, Cu, Mn, and Zn amounts of the dried apricot fruits changed to be between 10.4 and 80.1, 0.92 and 6.49, 0.97 and 8.27, and 2.96 and 12.0 µg/g, respectively [20]. Cranberry, *Lonicera caucasica*, “wild plum” and “blackberry” fruits contained 12–53 mg/100 g P, 287–585 mg/100 g K, 3.62–20.24 mg/100 g Na, 4–26.4 mg/100 g Ca, 7.97–12.30 mg/100 g Mg, 0.07–25.38 Fe mg/kg, 0.06–0.29 mg/kg Cu, 0.06–0.6 mg/kg Zn, and 0.05–1.96 mg/kg Mn [4]. In previous study, Dumbrava et al. [6] pointed out that dried brown raisins, dried dates, and dried figs contained 32,175 mg/kg, 6097 mg/kg, and 5730 mg/kg K, respectively. Dumbrava et al. [6] determined 2034 mg/kg Ca, and 1139 mg/kg P in dried figs. Phosphorus and K values of some wild fruits changed between 385 (“blackberry”) and 2538 (fig) to 6114 (medlar) and 18,613 mg/kg (wild strawberry), respectively [1]. Differences in the biogenic element contents of fruit samples may probably be due to the amount of skin content and pulp content of the fruits, and the element contents and climatic conditions of the soil where the fruits grow. Results showed some changes compared to the values of last studies [4, 20]. These changes can be due to fruit skin, fruit pulp, drying type, fruit type, ripening, climatic factors, and soil fertilizer.

**Table 2:** The moisture, macro, and micro element contents of wild and cultured fruits (dw)

Fruits	Moisture	P	K	Ca	Mg	Fe	Zn	Cu	Mn	B
	%	(mg kg^−1^)								
Cherry laurel	72.22 F	2050.98 B	9917.42 E	1583.94 D	765.26 C	15.64	0.88 G	2.65 F	8.24 B	27.00 C
Sour cherry	68.74 G	1853.38 C	12,440.60 C	1078.53 E	672.57 D	16.94	3.54 B	3.50 E	4.42 F	76.25 A
Cherry	74.12 DE	2130.19 B	14,360.12 B	1040.24 EF	663.92 D	60.13	2.28 DE	4.61 D	5.85 D	33.53 B
Pear	86.28 A	1632.74 D	9789.70 E	442.15 HI	442.99 G	34.48	1.70 F	7.76 B	5.19 E	10.96 F
Black nightshade	72.73 EF	4420.75 A	25,550.60 A	377.97 I	1852.04 A	15.61	8.97 A	9.40 A	12.59 A	6.57 G
Fragrant black grapes	73.12 EF	2144.60 B	12,245.89 C	458.43 HI	621.58 DE	10.58	0.84 G	6.32 C	2.71 I	24.52 D
Kaki persimmon	86.51 A	1296.04 E	7397.99 F	592.70 GHI	382.42 H	13.48	1.54 F	1.67 H	3.19 H	4.49 H
Black fig	80.91 C	1937.02 C	11,252.71 D	3637.15 B	1242.65 B	9.78	2.60 CD	3.62 E	5.62 DE	15.02 E
Strawberry tree	60.75 I	1326.92 E	5602.94 HI	821.41 FG	279.18 I	15.1	1.93 EF	2.39 FG	2.43 I	2.35 I
Hawthorn (yellow)	74.79 D	1284.41 E	11,564.14 CD	4330.89 A	546.57 F	14.55	2.79 C	1.86 GH	3.72 G	15.50 E
Elaeagnus	34.56 J	1267.36 E	6250.15 GH	662.78 GH	218.93 JK	5.59	3.61 B	1.87 GH	1.83 J	3.73 HI
Damask plum	64.09 H	1072.24 F	6819.74 FG	359.83 I	214.98 K	8.12	0.96 G	0.94 I	1.89 J	12.42 F
Japonica pear	84.02 B	1268.33 E	5696.64 HI	364.13 I	276.67 IJ	11.97	1.81 F	4.24 D	2.70 I	14.51 E
Myrtle (white)	70.30 G	1048.71 F	5212.77 I	3294.20 C	578.99 EF	5.97	1.55 F	2.68 F	7.72 C	6.75 G
Myrtle (black)	69.70 G	949.08 G	5216.27 I	1437.74 D	453.13 G	2.69	2.87 C	1.88 GH	1.92 J	3.76 HI

A. B: *p* < 0.01

### Principal component analysis

**Fig. 2:**
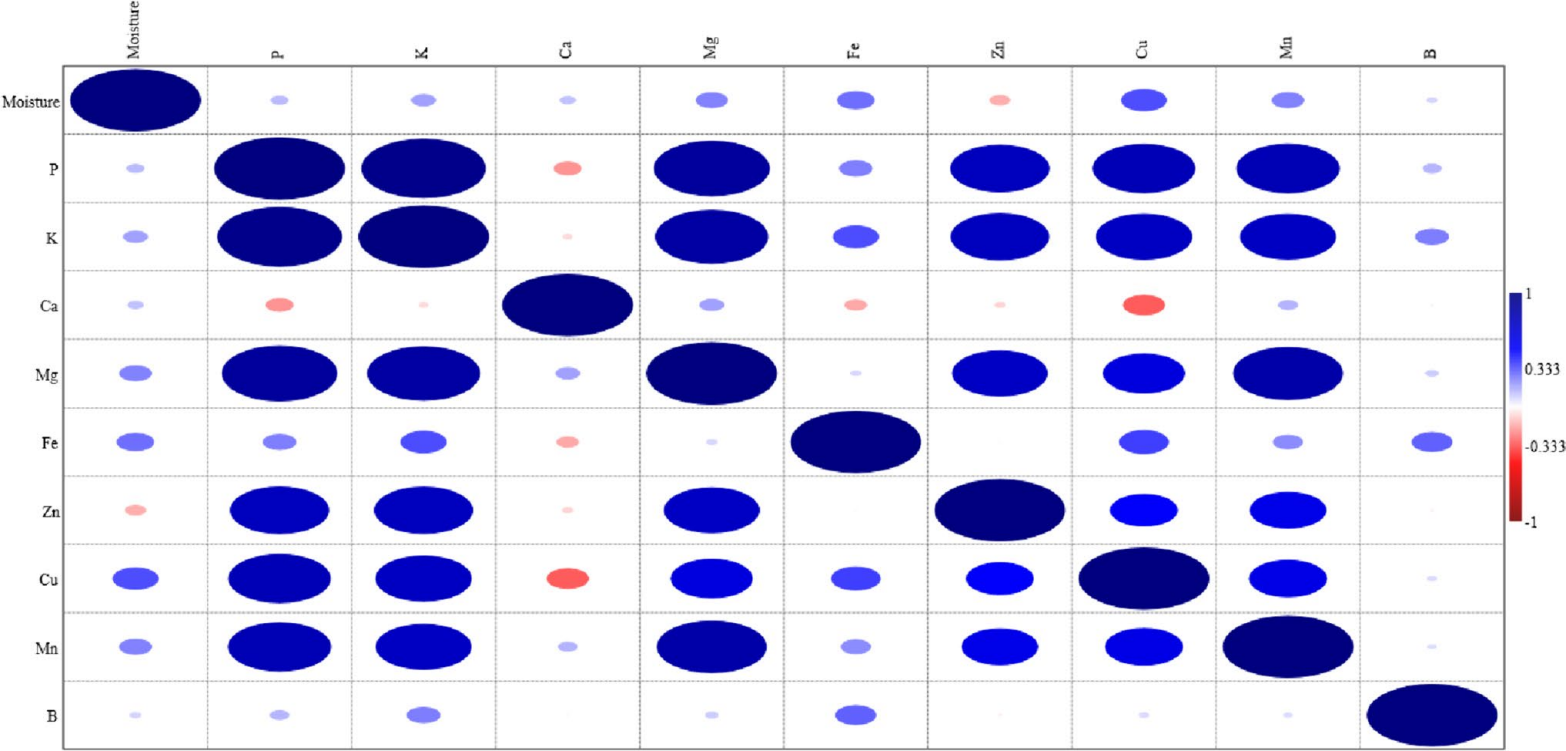
Fruit varieties (Black berry), sour cherry, cherry, pear, black grape, fragrant black grape, persimmon, black fig, strawberry tree, hawthorn (yellow), oleaster, damask plum, japanese pear, myrtle (white) and murt (black)), the Pearson correlation (*r*) between macro (P, K, Ca, and Mg) and micro (Fe, Zn, Mn, Cu, and B) nutrient contents and moisture contents

Fruit varieties (“Black nightshade,” “Sour cherry,” “Cherry,” “Pear,” “Fragrant black grape,” “Persimmon,” “Black fig,” “strawberry tree,” “Hawthorn (Yellow),” “elaeagnus,” “Damask plum,” “Japanese pear,” “Myrtle (White),” and “myrtle (Black)”) and the Pearson correlation (*r*) between macro (P, K, Ca, and Mg) and micro (Fe, Zn, Mn, Cu, and B) nutrient contents and moisture contents are given in Fig. 2. As can be seen by examining Fig. 2, it has been determined that there are positive relationships between moisture content and nutritional elements of fruit varieties. Although there are positive relationships between P contents of fruits and other nutritional elements, K (*r* = 0.951**), Mg (*r* = 0.885**), Zn (*r* = 0.758**), Cu (*r* = 0.783**), and Mn ( *r* = 0.787**) and its contents were reported to have significant and high strength relationships (*p* < 0.05, *r* > 0.70). It was determined that there were significant and highly strong positive relationships between the K contents of the varieties and their Mg (*r* = 0.863**), Zn (*r* = 0.753**), Cu (*r* = 0.736**), and Mn (*r* = 0.732**) contents have been made. While significant and moderately strong positive relationships (*p* < 0.05, *r* = 0.30–0.70) were determined between the Mg contents and Cu contents of the fruit varieties (*r* = 0.627**), Zn (*r* = 0.729**) and Mn (*r* = 0.840**) were determined. It has been revealed that there are significant and high-strong positive relationships between their contents and their contents. When looking at the Zn and Cu amounts of the fruits, this study determined that there were significant and moderately strong positive relationships between Zn contents and Cu (*r* = 0.515**) and Mn (*r* = 0.594**) contents, and between Cu contents and Mn contents (*r* = 0.594**).

## Conclusion

The element detected in the highest amounts in the fruits studied as a macro element was K, followed by P, Ca, and Mg. “Cherry laurel,” “sour cherry,” “cherry,” “pear,” “black nightshade,” “black fig,” and “hawthorn (yellow) fruits contain more K than other fruits. Also, “black fig,” “hawthorn (yellow),” and “myrtle (white)” fruits are richer in Ca than other fruits. The highest P content was detected in “black nightshade” fruits. In addition, “eleagnus” fruit is very rich in Zn. The highest Fe content was detected in “cherry laurel,” “sour cherry,” “cherry,” and “pear” fruits. The most abundant microelement detected in cultivated and wild fruits was B, followed by Fe, Mn, Cu, and Zn in decreasing order. In general, except for “white and black myrtle” fruits, it is thought that other fruits can be good sources of P, K and Fe.

## Data Availability

No datasets were generated or analysed during the current study.
